# Microchromosomes Exhibit Distinct Features of Vertebrate Chromosome Structure and Function with Underappreciated Ramifications for Genome Evolution

**DOI:** 10.1093/molbev/msaa253

**Published:** 2020-09-28

**Authors:** Blair W Perry, Drew R Schield, Richard H Adams, Todd A Castoe

**Affiliations:** Department of Biology, University of Texas at Arlington, Arlington, TX

**Keywords:** Hi-C sequencing, chromosome territories, chromatin, gene regulation

## Abstract

Microchromosomes are common yet poorly understood components of many vertebrate genomes. Recent studies have revealed that microchromosomes contain a high density of genes and possess other distinct characteristics compared with macrochromosomes. Whether distinctive characteristics of microchromosomes extend to features of genome structure and organization, however, remains an open question. Here, we analyze Hi-C sequencing data from multiple vertebrate lineages and show that microchromosomes exhibit consistently high degrees of interchromosomal interaction (particularly with other microchromosomes), appear to be colocalized to a common central nuclear territory, and are comprised of a higher proportion of open chromatin than macrochromosomes. These findings highlight an unappreciated level of diversity in vertebrate genome structure and function, and raise important questions regarding the evolutionary origins and ramifications of microchromosomes and the genes that they house.

The 3D organization and interactions of the genome play fundamental roles in gene regulation and genome function (Cremer et al. 1993; Cremer and Cremer 2001). Advances in functional genomics approaches, such as Hi-C sequencing (Lieberman-Aiden et al. 2009), have broadened our understanding of 3D genomic interactions and organization in the nucleus, including how chromatin loops coordinate the regulation of genes and how chromosomes form discrete chromosome territories within the nucleus (Cremer et al. 1993; Cremer and Cremer 2001; Bolzer et al. 2005). Most studies of 3D genome organization and structure have focused on mammalian genomes that are exclusively comprised of macrochromosomes (Cremer et al. 1993; Kurz et al. 1996; Zink et al. 1998; Cremer and Cremer 2001). However, many non-mammalian vertebrates possess microchromosomes—nuclear chromosomes generally <30 Mb in length—in addition to macrochromosomes (Ohno et al. 1969; Burt 2002; Zhou and Gui 2002; Consortium ICGS 2004; Axelsson et al. 2005; Schield et al. 2019). Microchromosome number is variable across vertebrates, ranging from 0 in macrochromosome-only lineages to >40 in other lineages (Deakin and Ezaz 2019; O’Connor et al. 2019). Vertebrate microchromosomes consistently exhibit many distinct features across lineages, including high gene density, low transposable element content, and high rates of recombination (Consortium ICGS 2004; Backström et al. 2010; Schield et al. 2019; 2020), and represent a functionally and evolutionarily unique fraction of the genomes of many vertebrates. However, it remains largely unknown how 3D genomic features manifest in nuclei of vertebrates containing both macro- and microchromosomes.

Recent Hi-C studies of vertebrates with microchromosomes have provided increasing evidence for distinct features of microchromosome organization and function. A study of the Prairie Rattlesnake (*Crotalus viridis*) found that microchromosomes exhibit higher degrees of interaction with other chromosomes than expected based on chromosome size (Schield et al. 2019). A similar trend was observed in chicken erythrocytes (*Gallus gallus*) (Fishman et al. 2019). This study also inferred AB compartments across the chicken genome, which broadly correspond to regions of open (A compartment) and closed (B compartment) chromatin (Lieberman-Aiden et al. 2009), and showed that microchromosomes exhibit a higher proportion of A compartment regions than macrochromosomes (Fishman et al. 2019). Together, these studies suggest that microchromosomes may be functionally and organizationally distinct compared with macrochromosomes. The extent to which these patterns represent universal characteristics of microchromosomes remains unexplored, and their evolutionary causes and ramifications largely unconsidered.

Here, we use recently published chromosome-level genome assemblies and Hi-C data sets for representatives of multiple vertebrate lineages to infer patterns of 3D interaction and organization of genomes that possess both macro- and microchromosomes. Based on these data, we demonstrate that high interchromosomal interaction and enrichment for A compartment regions are likely ubiquitous features of vertebrate microchromosomes, and find support for previous suggestions that microchromosomes co-inhabit the center of the nucleus. Collectively, these findings suggest that vertebrate genomes with microchromosomes may structurally, functionally, and evolutionarily operate in fundamentally distinct ways compared with macrochromosome-only genomes. This conclusion highlights the largely unexplored evolutionary relevance of the presence/absence of microchromosomes across vertebrate lineages, and the relevance of genes being encoded on microchromosomes.

## Results

**Fig. 1.**
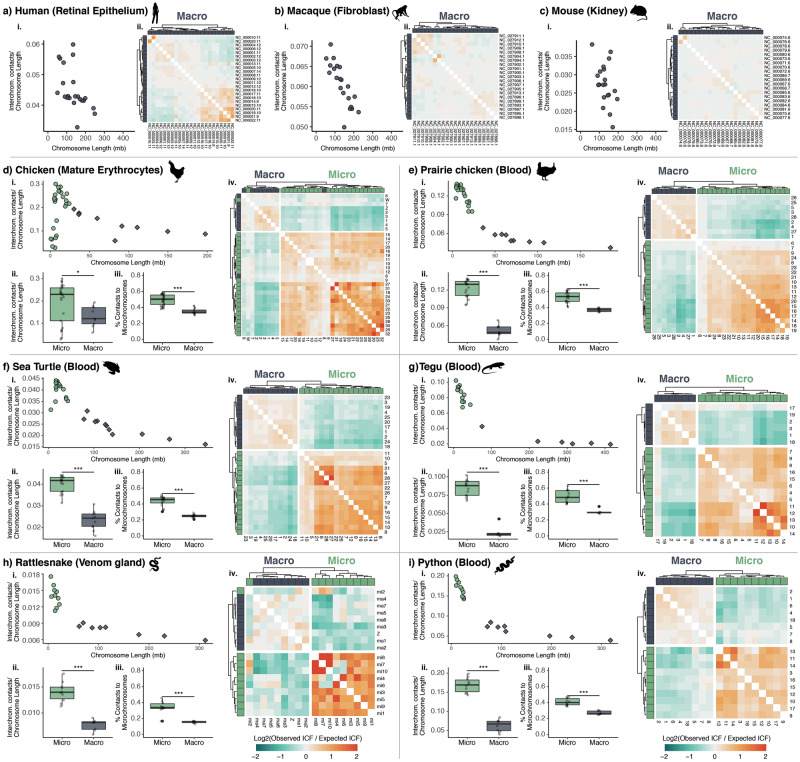
Microchromosomes exhibit elevated interchromosomal contact frequencies and interact preferentially with other microchromosomes. (*a*_i_–*i*_i_) Sums of interchromosomal contact frequencies per chromosome normalized by chromosome length plotted over chromosome length. (*d*_ii_–*i*_ii_) Comparisons of interchromosomal contact frequency normalized by chromosome length for macro and microchromosomes (*P-value < 0.05, ***P-value < 0.001, Student’s *t*-test). (*d*_iii_–*i*_iii_) Comparison of the proportion of interchromosomal contacts that involve a microchromosome for macrochromosomes and microchromosomes (*** denotes *P* < 0.001, Student’s *t*-test). (*a*_ii_–*c*_ii_, *d*_iv_–*i*_iv_) Heatmaps of the ratio of observed to expected interchromosomal contact frequency (ICF) between all chromosome pairs, with hierarchical clustering and chromosome type annotated above and to the left of each heatmap.

Our analyses of Hi-C data indicate that, for all species analyzed (supplementary tables 1 and 2, Supplementary Material online), interchromosomal contact frequency (ICF) generally increases as chromosome size decreases (fig. 1*a*_i_–*i*_i_). Microchromosomes therefore exhibit a higher degree of interchromosomal interaction, with all non-mammalian species exhibiting a significantly higher degree of interchromosomal interaction in microchromosomes than in macrochromosomes (fig. 1*d*_ii_–*i*_ii_). Interestingly, in the chicken, which possesses the smallest microchromosomes among all species we analyzed, there is an apparent inflection point in chromosome size at which interchromosomal activity begins to decrease as chromosome size continues to decrease (fig. 1*d*_i_, supplementary fig. 1, Supplementary Material online). This pattern is apparent in all three chicken tissues analyzed, and less pronounced inflection points near the smallest microchromosomes in the Prairie Chicken (fig. 1*e*_i_) and Sea Turtle (fig. 1*f*_i_).

To further investigate patterns of interchromosomal contacts between macrochromosomes and microchromosomes, we compared empirical ICFs to ICFs predicted by a null model assuming uniform interactions between chromosomes, following (Zhang et al. 2012). In all non-mammalian species, we find an excess of ICFs between microchromosome pairs and fewer than expected ICFs between macrochromosomes and microchromosomes (fig. 1*d*_iv_–*i*_iv_). Hierarchical clustering of chromosomes based on observed over expected ICFs distinguishes macrochromosomes from microchromosomes in nearly all species and tissues, with a small number of exceptions in the rattlesnake (fig. 1_iv_) and the three chicken tissues analyzed (supplementary fig. 1, Supplementary Material online).

**Fig. 2.**
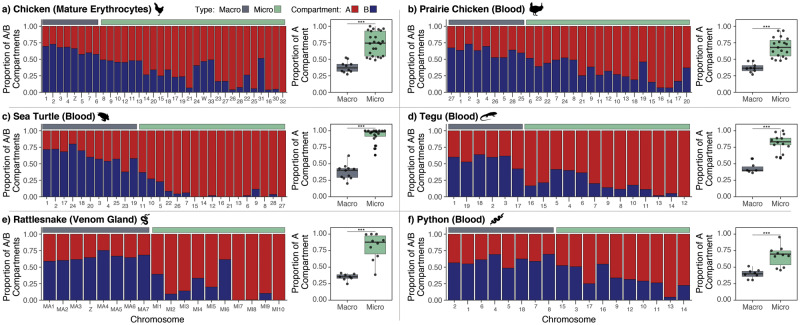
Microchromosomes are enriched for the A compartment. Bar plots indicate the proportion of 50-kb bins for each chromosome that were determined to be A (red) and B (blue) compartment. In all species, microchromosomes exhibit a higher proportion of A compartment bins than macrochromosomes (boxplots on right; *** denotes *P* < 0.001, Student’s *t*-test).

For all species possessing microchromosomes, we inferred AB compartments based on patterns of ICFs at 50 kb resolution between all chromosomes and binned measures of GC content. We find that microchromosomes in all species are comprised of a significantly higher proportion of A compartment regions compared with macrochromosomes, which are predominately comprised of B compartment regions (fig. 2, supplementary fig. 2, Supplementary Material online).

**Fig. 3.**
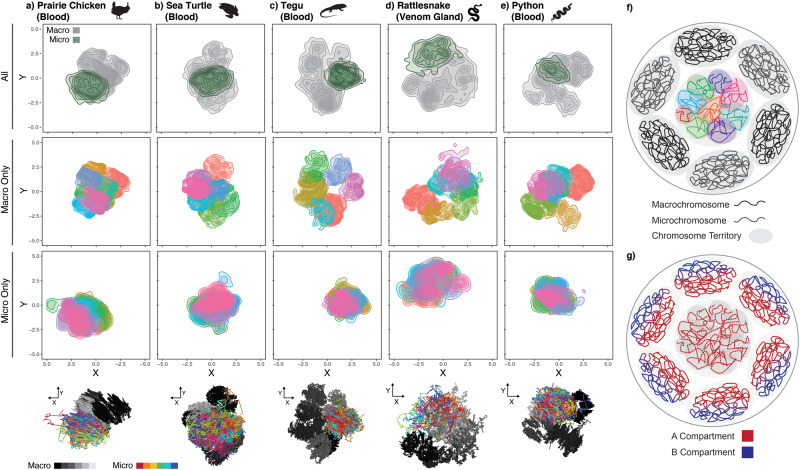
Microchromosomes are likely co-localized in the 3D nucleus. (*a*–*e*) 3D interpretations of Hi-C interaction data shown as 2D point density plots from three distinct orientations for all chromosomes, macrochromosomes only, and microchromosomes only. For macro and micro only plots, different colors represent different chromosomes. Shown at the bottom are 3D interpretations of all chromosomes, with macrochromosomes in grayscale and microchromosomes in color. Additional orientations for each species are available in supplementary figures 4–8, Supplementary Material online. (*f*–*g*) Cartoon representations of a nucleus illustrating the hypotheses that (*f*) microchromosomes are centrally located in the nucleus and collectively inhabit a “microchromosome territory” and (*g*) that of spatial organization of A and B compartments in a nucleus containing A-rich microchromosomes.

Genome-wide heatmaps of binned Hi-C contact frequency and 3D interpretations of interaction data both show evidence of well-defined chromosome territories for macrochromosomes (fig. 3, supplementary figs. 3–8, Supplementary Material online). For microchromosomes, contact frequency heatmaps show elevated levels of intrachromosomal interaction (supplementary fig. 3, Supplementary Material online), and show an elevated degree of microchromosome–microchromosome interaction. Furthermore, this high degree of microchromosome interaction results in a lack of obvious spatial distinction between microchromosomes in 3D interpretations of Hi-C interaction data, and independent microchromosome territories are not well defined (fig. 3, supplementary figs. 3–8, Supplementary Material online). Although 3D interpretations of Hi-C data should not be directly interpreted as biologically accurate models of the nucleus, they do provide fairly robust inferences regarding the degree of isolation of chromosomes based on patterns of 2D interaction. Note that 3D models were not generated for the three chicken tissues due to the data for several microchromosomes being too sparse to generate intrachromosomal contact maps at necessary resolution.

## Discussion

Using Hi-C contact data from diverse vertebrate lineages, we demonstrate that microchromosomes consistently exhibit an elevated degree of interchromosomal interactivity compared with that of macrochromosomes. This pattern of elevated inter-chromosomal interaction for microchromosomes is consistent with previous studies of single species (chicken [Fishman et al. 2019], and rattlesnake [Schield et al. 2019]), and our expanded sampling indicate that these patterns are likely remarkably consistent across diverse vertebrate lineages. We consistently find that the high magnitude of microchromosome interactivity is dominated by microchromosome-to-microchromosome interactions, and additionally show that microchromosomes are consistently enriched for, and in many cases comprised almost exclusively of, A compartment regions. These findings emphasize the unique structural and functional features of vertebrate microchromosomes, and raise interesting questions about the relationships between microchromosome structure and genome function and organization.

Previous microscopy studies have suggested that bird microchromosomes inhabit the center of the nucleus with macrochromosomes arranged around them at the nuclear periphery (Habermann et al. 2001; Skinner et al. 2009; Berchtold et al. 2011). Similar studies have not yet, however, been conducted for other species with microchromosomes (i.e., fish, non-avian reptiles), and the degree to which this chromosomal arrangement is conserved across vertebrates with microchromosomes remains unknown. Our findings of consistently elevated microchromosome–microchromosome interactions are consistent with a model in which microchromosomes are localized in the center of the nucleus across diverse vertebrate lineages. This arrangement of microchromosomes is also supported by our inference that microchromosomes are primarily comprised of A compartment (open chromatin) regions, which tend to be concentrated at the center of the nucleus (Kosak et al. 2007; Misteli 2007). Taken together, our Hi-C based inferences and previous studies tentatively support a model of nuclear organization in which A-rich microchromosomes occupy the center of the nucleus, surrounded by A-rich regions of macrochromosomes that inhabit the nuclear periphery (fig. 3*g*). Interestingly, somewhat analogous examples exist in insect chromosomes (e.g., *Drosophila* dot chromosome), in which these chromosomes with distinct compositional characteristics (heterochromatic, gene dense, transposon-rich) occupy distinct regions of the nucleus (Riddle and Elgin 2018), implying broad links between nuclear chromosome organization and chromosome composition, structure and function. Future studies that utilize 3D fluorescence in situ hybridization for multiple vertebrates with microchromosomes would be particularly valuable for testing our hypotheses for nuclear organization, and the degree to which it is conserved across species and cell types.

Available evidence suggests that microchromosomes collectively exhibit features that are distinct from typical macrochromosomes, in that they are closely associated in the nucleus and interact more frequently with other microchromosomes than to macrochromosomes. This argues for the presence of a microchromosome-specific territory in the nucleus that features a higher degree of interchromosomal interaction than typically observed for macrochromosomes (fig. 3*f*). However, the degree to which microchromosomes inhabit well-defined individual territories within this encompassing microchromosome territory remains an open question; it is possible that the lack of defined microchromosome territories in our 3D interpretations of Hi-C data may result from variable positioning of microchromosomes across sampled cells (i.e., a merged “average” of relative position). It also remains an open question how such an arrangement of microchromosomes may influence the formation and position of the nucleolus in the nucleus. Regardless, the high degree of interaction among microchromosomes raises the possibility of interchromosomal regulatory interactions between microchromosomes, a phenomenon thought to be rare in macrochromosomes (Bashkirova and Lomvardas 2019; Maass et al. 2019) that should be explored further in microchromosomes.

Although our findings show notably similar characteristics between microchromosomes of multiple vertebrate lineages, it is worth noting that our current sampling is remarkably sparse in the context of vertebrate diversity, and lacks representatives from several important lineages that also possess microchromosomes (i.e., fish) for which Hi-C contact information data are not currently available. Although we do observe consistent patterns across many of the tissue and cell types sampled here (whole blood, venom gland, erythrocytes) that may represent common features of microchromosome biology and organization, we expect variation and exceptions to these patterns to exist in various cell types, tissues, and developmental stages within species. Indeed, we observed evidence of variation in interchromosomal contact patterns when various chicken cell types are compared, with some of these variations being particularly distinct in chicken embryonic fibroblast cells (supplementary fig. 1, Supplementary Material online). The degree to which patterns of microchromosome interaction and structure observed here are broadly present and/or consistent across the full diversity of vertebrate lineages, tissue, and cell types therefore remains an open question for future studies, as additional data for diverse vertebrates becomes available.

A major consideration emphasized by our findings is how unique features of microchromosomes may affect the evolution of genes housed on microchromosomes. Unlike macrochromosomes, microchromosomes tend to share a common nuclear territory, and have high levels of interchromosomal interaction, and consist of mainly A compartment active chromatin. Intriguingly, despite this unusually high level of interchromosomal interaction, which may suggest functional interactions among microchromosomes, they segregate independently and consistently exhibit among the highest genome-wide recombination rates (Consortium ICGS 2004; Backström et al. 2010; Schield et al. 2020). This has profound implications for the evolution of genes on microchromosomes, and suggests that the rate and efficiency of selection, and the effects of drift, would be distinct on microchromosomes compared with macrochromosomes. For example, high recombination rates in microchromosomes would be very effective at breaking down linkage disequilibrium, breaking associations among selected alleles, and thereby increasing the efficacy of selection. These features suggest that microchromosomes possess ideal characteristics for housing genes underlying adaptation. Anecdotal support for this comes from the Prairie Rattlesnake genome, in which microchromosomes contain the majority of important venom genes, which are generally known to be under strong local selection (Mackessy 2010; Casewell et al. 2013; Schield et al. 2019), although more extensive systematic studies of additional vertebrate lineages would be necessary to test hypotheses for the special relevance of microchromosomes in adaptation. Continued accumulation of chromosome-level genome resources for diverse vertebrates will provide new opportunities to test hypotheses related to the roles of microchromosomes in genome evolution, investigate the relevance of genes and gene families being located on microchromosomes, and elucidate the factors that drive shifts from macrochromosome-only systems to those containing both chromosome types.

## Materials and Methods

Hi-C data were downloaded from the NCBI Sequence Read Archive for the Prairie Rattlesnake (*C. viridis*), Burmese Python (*Python bivittatus*), Argentine Black and White Tegu (*Salvator merianae*), Green Sea Turtle (*Chelonia mydas*), Greater Prairie Chicken (*Tympanuchus cupido*), chicken (*G. gallus*), Rhesus Macaque (*Macaca mulatta*), Patski Mouse (*Mus musculus* × *Mus spretus*), and human (*Homo sapiens*). (See supplementary table 1, Supplementary Material online for details.) Hi-C reads for each species were mapped to genome assemblies and processed using the Juicer pipeline (Durand, Shamim, et al. 2016). For each species, inter and intrachromosomal contact matrices were extracted from the resulting Hi-C map using the dump command in Juicer Tools v1.9.9 at 50 kb, 100 kb, and 1 Mb resolutions using KR-normalization and only reads that mapped with MAPQ > 30. The size at which a chromosome is designated a microchromosome is not well defined, and most previous studies have defined microchromosomes and macrochromosomes largely based on visual dichotomies apparent in chromosome squashes (e.g., Habermann et al. 2001). In this study, avian microchromosomes were defined as chromosomes <30 Mb. Visual inspection of linear chromosome length for non-avian reptiles revealed a more apparent natural break between larger and smaller chromosomes ∼50 Mb, and we therefore defined chromosomes shorter than this as microchromosomes. Downstream analyses of observed versus expected ICFs (described below) lend support to this breakpoint, as chromosomes defined herein as macrochromosomes and microchromosomes based on these criteria cluster strongly with others of the same type, with few exceptions (see fig. 1).

The sum of all interchromosomal contacts per chromosome was divided by chromosome length to produce a relative measure of interchromosomal contact density per chromosome, and the relationship between this normalized contact frequency and chromosome length was tested using linear regression in R (R Core Team 2018). Differences between macrochromosome and microchromosome ICFs were tested using Student’s *t*-tests. Observed contact frequencies were compared with the expected ICF for each chromosome pair assuming uniform interactions between chromosomes following (Zhang et al. 2012). The log2 ratio of observed over expected ICF was plotted as a heatmap in R using pheatmap v1.0.12 (https://github.com/raivokolde/pheatmap). Heatmaps of Hi-C contact frequency were generated with Juicebox (Durand, Robinson, et al. 2016).

miniMDS (Rieber and Mahony 2017) was used to generate 3D interpretations of Hi-C data using 1 Mb resolution interchromosomal contact data and 50 kb resolution intrachromosomal contact data. miniMDS was run using full partitioning with minimum partition size 0.08 and the default smoothing parameter. The resulting 3D models were visualized using Mayavi (Ramachandran and Varoquaux 2011). Note that Hi-C data for the three chicken tissues were too sparse to generate 50 kb intrachromosomal contact maps for input into miniMDS, and therefore these samples were excluded from 3D modeling.

Juicer Hi-C matrices were converted to the cooler format (Abdennur and Mirny 2020) at 50 kb resolution using hic2cool v0.8.3 (https://github.com/4dn-dcic/hic2cool) and normalized using “balance” within the cooler CLI package v0.8.7 (Abdennur and Mirny 2020). GC content was measured in 50 kb bins using the “nuc” program within bedtools v2.29.0 (Quinlan and Hall 2010). AB compartments were determined with “call-compartments” within cooltools v0.3.2 (https://github.com/mirnylab/cooltools) using trans (interchromosomal) contacts and binned measures of GC content as the reference track. The proportion of A compartment regions per chromosome was calculated as the number of 50-kb bins determined to belong to the A compartment divided by the total number of bins representing the chromosome and plotted in R. A Student’s *t*-test was used to test for enrichment of A compartments on microchromosomes.

## Supplementary Material

Supplementary data are available at *Molecular Biology and Evolution* online.

## Supplementary Material

msaa253_Supplementary_Data

## References

[msaa253-B1] Abdennur N , Mirny LA. 2020. Cooler: scalable storage for Hi-C data and other genomically labeled arrays. Bioinformatics 36(1):311–316. 31290943 10.1093/bioinformatics/btz540 PMC8205516

[msaa253-B2] Axelsson E , Webster MT, Smith NGC, Burt DW, Ellegren H. 2005. Comparison of the chicken and turkey genomes reveals a higher rate of nucleotide divergence on microchromosomes than macrochromosomes. Genome Res. 15(1):120–125. 15590944 10.1101/gr.3021305 PMC540272

[msaa253-B3] Backström N , Forstmeier W, Schielzeth H, Mellenius H, Nam K, Bolund E, Webster MT, Ost T, Schneider M, Kempenaers B, et al 2010. The recombination landscape of the zebra finch *Taeniopygia guttata* genome. Genome Res. 20(4):485–495., 20357052 10.1101/gr.101410.109 PMC2847751

[msaa253-B4] Bashkirova E , Lomvardas S. 2019. Olfactory receptor genes make the case for inter-chromosomal interactions. Curr Opin Genet Dev. 55:106–113. 31491591 10.1016/j.gde.2019.07.004 PMC6759391

[msaa253-B5] Berchtold D , Fesser S, Bachmann G, Kaiser A, Eilert J-C, Frohns F, Sadoni N, Muck J, Kremmer E, Eick D, et al 2011. Nuclei of chicken neurons in tissues and three-dimensional cell cultures are organized into distinct radial zones. Chromosome Res. 19(2):165–182. 21249442 10.1007/s10577-010-9182-3

[msaa253-B6] Bolzer A , Kreth G, Solovei I, Koehler D, Saracoglu K, Fauth C, Müller S, Eils R, Cremer C, Speicher MR, et al 2005. Three-dimensional maps of all chromosomes in human male fibroblast nuclei and prometaphase rosettes. PLoS Biol. 3(5):e157. 15839726 10.1371/journal.pbio.0030157 PMC1084335

[msaa253-B7] Burt DW. 2002. Origin and evolution of avian microchromosomes. Cytogenet Genome Res. 96(1–4):97–112. 12438785 10.1159/000063018

[msaa253-B8] Casewell NR , Wuster W, Vonk FJ, Harrison RA, Fry BG. 2013. Complex cocktails: the evolutionary novelty of venoms. Trends Ecol Evol. 28(4):219–229. 23219381 10.1016/j.tree.2012.10.020

[msaa253-B9] Consortium ICGS. 2004. Sequence and comparative analysis of the chicken genome provide unique perspectives on vertebrate evolution. Nature 432:695–716. 15592404 10.1038/nature03154

[msaa253-B10] Cremer T , Cremer C. 2001. Chromosome territories, nuclear architecture and gene regulation in mammalian cells. Nat Rev Genet. 2(4):292–301. 11283701 10.1038/35066075

[msaa253-B11] Cremer T , Kurz A, Zirbel R, Dietzel S, Rinke B, Schröck E, Speicher MR, Mathieu U, Jauch A, Emmerich P. 1993. Role of chromosome territories in the functional compartmentalization of the cell nucleus. In: Cold spring harbor symposia on quantitative biology. Vol. 58. Cold Spring Harbor, NY: Cold Spring Harbor Laboratory Press. p. 777–792. 7525149 10.1101/sqb.1993.058.01.085

[msaa253-B12] Deakin JE , Ezaz T. 2019. Understanding the evolution of reptile chromosomes through applications of combined cytogenetics and genomics approaches. Cytogenet Genome Res. 157(1–2):7–20. 30645998 10.1159/000495974

[msaa253-B13] Durand NC , Robinson JT, Shamim MS, Machol I, Mesirov JP, Lander ES, Aiden EL. 2016. Juicebox provides a visualization system for Hi-C contact maps with unlimited zoom. Cell Syst. 3(1):99–101. 27467250 10.1016/j.cels.2015.07.012 PMC5596920

[msaa253-B14] Durand NC , Shamim MS, Machol I, Rao SSP, Huntley MH, Lander ES, Aiden EL. 2016. Juicer provides a one-click system for analyzing loop-resolution Hi-C experiments. Cell Syst. 3(1):95–98. 27467249 10.1016/j.cels.2016.07.002 PMC5846465

[msaa253-B15] Fishman V , Battulin N, Nuriddinov M, Maslova A, Zlotina A, Strunov A, Chervyakova D, Korablev A, Serov O, Krasikova A. 2019. 3D organization of chicken genome demonstrates evolutionary conservation of topologically associated domains and highlights unique architecture of erythrocytes’ chromatin. Nucleic Acids Res. 47(2):648–665. 30418618 10.1093/nar/gky1103 PMC6344868

[msaa253-B16] Habermann FA , Cremer M, Walter J, Kreth G, von Hase J, Bauer K, Wienberg J, Cremer C, Cremer T, Solovei I. 2001. Arrangements of macro-and microchromosomes in chicken cells. Chromosom Res. 9(7):569–584. 10.1023/a:1012447318535 11721954

[msaa253-B17] Kosak ST , Scalzo D, Alworth SV, Li F, Palmer S, Enver T, Lee JSJ, Groudine M. 2007. Coordinate gene regulation during hematopoiesis is related to genomic organization. PLoS Biol. 5(11):e309. 18031200 10.1371/journal.pbio.0050309 PMC2080650

[msaa253-B18] Kurz A , Lampel S, Nickolenko JE, Bradl J, Benner A, Zirbel RM, Cremer T, Lichter P. 1996. Active and inactive genes localize preferentially in the periphery of chromosome territories. J Cell Biol. 135(5):1195–1205. 8947544 10.1083/jcb.135.5.1195 PMC2121085

[msaa253-B19] Lieberman-Aiden E , van Berkum NL, Williams L, Imakaev M, Ragoczy T, Telling A, Amit I, Lajoie BR, Sabo PJ, Dorschner MO, et al 2009. Comprehensive mapping of long-range interactions reveals folding principles of the human genome. Science (80-) 326(5950):289–293. 10.1126/science.1181369 PMC2858594 19815776

[msaa253-B20] Maass PG , Barutcu AR, Rinn JL. 2019. Interchromosomal interactions: a genomic love story of kissing chromosomes. J Cell Biol. 218(1):27–38. 30181316 10.1083/jcb.201806052 PMC6314556

[msaa253-B21] Mackessy SP. 2010. Evolutionary trends in venom composition in the western rattlesnakes (*Crotalus viridis* sensu lato): toxicity vs. tenderizers. Toxicon 55:1463–1474. 20227433 10.1016/j.toxicon.2010.02.028

[msaa253-B22] Misteli T. 2007. Beyond the sequence: cellular organization of genome function. Cell 128(4):787–800. 17320514 10.1016/j.cell.2007.01.028

[msaa253-B23] O’Connor RE , Kiazim L, Skinner B, Fonseka G, Joseph S, Jennings R, Larkin DM, Griffin DK. 2019. Patterns of microchromosome organization remain highly conserved throughout avian evolution. Chromosoma 128:21–29. 30448925 10.1007/s00412-018-0685-6 PMC6394684

[msaa253-B24] Ohno S , Muramoto J, Stenius C, Christian L, Kittrell WA, Atkin NB. 1969. Microchromosomes in holocephalian, chondrostean and holostean fishes. Chromosoma 26(1):35–40. 5799423 10.1007/BF00319498

[msaa253-B25] Quinlan AR , Hall IM. 2010. BEDTools: a flexible suite of utilities for comparing genomic features. Bioinformatics 26(6):841–842. 20110278 10.1093/bioinformatics/btq033 PMC2832824

[msaa253-B26] Ramachandran P , Varoquaux G. 2011. Mayavi: 3D visualization of scientific data. Comput Sci Eng. 13(2):40–51.

[msaa253-B27] Riddle NC , Elgin SCR. 2018. The Drosophila dot chromosome: where genes flourish amidst repeats. Genetics 210(3):757–772. 30401762 10.1534/genetics.118.301146 PMC6218221

[msaa253-B28] Rieber L , Mahony S. 2017. miniMDS: 3D structural inference from high-resolution Hi-C data. Bioinformatics 33(14):i261–i266. 28882003 10.1093/bioinformatics/btx271 PMC5870652

[msaa253-B29] Schield DR , Card DC, Hales NR, Perry BW, Pasquesi GM, Blackmon H, Adams RH, Corbin AB, Smith CF, Ramesh B, et al 2019. The origins and evolution of chromosomes, dosage compensation, and mechanisms underlying venom regulation in snakes. Genome Res. 29(4):590–601., 30898880 10.1101/gr.240952.118 PMC6442385

[msaa253-B30] Schield DR , Pasquesi GIM, Perry BW, Adams RH, Nikolakis ZL, Westfall AK, Orton RW, Meik JM, Mackessy SP, Castoe TA. 2020. Snake recombination landscapes are concentrated in functional regions despite PRDM9. Mol Biol Evol. 37(5):1272–1294. 31926008 10.1093/molbev/msaa003

[msaa253-B31] Skinner BM , Völker M, Ellis M, Griffin DK. 2009. An appraisal of nuclear organisation in interphase embryonic fibroblasts of chicken, turkey and duck. Cytogenet Genome Res. 126(1–2):156–164. 20016165 10.1159/000245915

[msaa253-B32] R Core Team. 2018. R: a language and environment for statistical computing. Available from: https://www.R-project.org/. Accessed June 2020.

[msaa253-B33] Zhang Y , McCord RP, Ho Y-J, Lajoie BR, Hildebrand DG, Simon AC, Becker MS, Alt FW, Dekker J. 2012. Spatial organization of the mouse genome and its role in recurrent chromosomal translocations. Cell 148(5):908–921. 22341456 10.1016/j.cell.2012.02.002 PMC3320767

[msaa253-B34] Zhou L , Gui JF. 2002. Karyotypic diversity in polyploid gibel carp, *Carassius auratus gibelio* Bloch. Genetica 115(2):223–232. 12403177 10.1023/a:1020102409270

[msaa253-B35] Zink D , Cremer T, Saffrich R, Fischer R, Trendelenburg MF, Ansorge W, Stelzer EHK. 1998. Structure and dynamics of human interphase chromosome territories in vivo. Hum Genet. 102(2):241–251. 9521598 10.1007/s004390050686

