# Supplementary material for: Microchromosomes Exhibit Distinct Features of Vertebrate Chromosome Structure and Function with Underappreciated Ramifications for Genome Evolution
Source: Mol Biol Evol. 2020 Sep 28;38(3):904–10. doi: 10.1093/molbev/msaa253 (PMC7947875; doi:10.1093/molbev/msaa253)
Supplement: msaa253_Supplementary_Data [file msaa253_supplementary_data.pdf]

Supplementary Table 1. Hi-C datasets used in this study.

Species	Common Name	Tissue	Microchromosomes	Source	NCBI Accession
Homo sapiens	Human	Retinal Epithelium	No	(Rao et al. 2014)	GEO: GSE63525
Mus musculus x Mus spretus	Mouse (Patski cell line)	Kidney	No	(Darrow et al. 2016)	GEO: GSE71831
Macaca mulatta	Rhesus Macaque	Fibroblast	No	(Darrow et al. 2016)	GEO: GSE71831
Gallus gallus	Chicken	Mature Erythrocytes	**Yes**	(Fishman et al. 2018)	BioSample: SAMN06555414, SAMN06555414
		Immature Erythrocytes	**Yes**	(Fishman et al. 2018)	BioSample: SAMN10291560, SAMN10291559
		Embryonic Fibroblasts	**Yes**	(Fishman et al. 2018)	BioSample: SAMN06555417, SAMN06555416
Tympanuchus cupido	Greater Prairie Chicken	Blood	**Yes**	(Johnson et al.; Dudchenko et al. 2017; Dudchenko et al. 2018)	BioSample: SAMN10973758
Chelonia mydas	Green Sea Turtle	Blood	**Yes**	(Wang et al. 2013; Dudchenko et al. 2017; Dudchenko et al. 2018)	BioSample: SAMN10973717
Salvator merianae	Argentine Black and White Tegu	Blood	**Yes**	(Dudchenko et al. 2017; Dudchenko et al. 2018; Roscito et al. 2018)	BioSample: SAMN10973771
Crotalus viridis	Prairie Rattlesnake	Venom gland	**Yes**	(Schield et al. 2019)	BioSample: SAMN07738522
Python bivittatus	Burmese Python	Blood	**Yes**	(Castoe et al. 2013; Dudchenko et al. 2017; Dudchenko et al. 2018)	BioSample: SAMN10973752

Supplementary Table 2. Hi-C mapping and contact statistics output from Juicer Hi-C analysis pipeline.

	Human (Retinal Epithelium)	Mouse (Kidney)	Macaque (Fibroblast)	Chicken (Mature Erythrocytes)	Chicken (Immature Erythrocytes)	Chicken (Embryonic Fibroblasts)	Prairie Chicken (Blood)	Sea Turtle (Blood)	Tegu (Blood)	Rattlesnake (Venom Gland)	Python (Blood)
**Sequenced Read Pairs**	626,007,610	580,083,428	701,157,628	261,175,632	358,570,027	276,677,613	118,671,947	135,271,653	140,870,003	195,378,673	274,127,665
**Normal Paired (% Sequenced Reads)**	305,144,884 (48.74%)	379,398,498 (65.40%)	579,963,191 (82.72%)	173,992,150 (66.62%)	174,107,133 (48.56%)	217,640,966 (78.66%)	100,090,013 (84.34%)	49,344,486 (36.48%)	73,786,879 (52.38%)	124,506,379 (63.73%)	129,656,423 (47.30%)
**Chimeric Paired (% Sequenced Reads)**	242,920,080 (38.80%)	0 (0.00%)	81,842,940 (11.67%)	77,562,745 (29.70%)	146,084,058 (40.74%)	44,525,771 (16.09%)	10,283,192 (8.67%)	63,913,102 (47.25%)	56,100,941 (39.82%)	44,190,474 (22.62%)	123,903,833 (45.20%)
**Chimeric Ambiguous (% Sequenced Reads)**	71,331,847 (11.39%)	0 (0.00%)	18,541,357 (2.64%)	3,632,523 (1.39%)	8,650,141 (2.41%)	2,650,906 (0.96%)	1,372,378 (1.16%)	18,123,962 (13.40%)	7,560,451 (5.37%)	18,225,522 (9.33%)	16,051,407 (5.86%)
**Unmapped (% Sequenced Reads)**	6,610,799 (1.06%)	200,684,930 (34.60%)	20,810,140 (2.97%)	5,988,214 (2.29%)	29,728,695 (8.29%)	11,859,970 (4.29%)	6,926,364 (5.84%)	3,890,103 (2.88%)	3,421,732 (2.43%)	8,456,298 (4.33%)	4,516,002 (1.65%)
**Alignable (% Sequenced Reads)**	548,064,964 (87.55%)	379,398,498 (65.40%)	661,806,131 (94.39%)	251,554,895 (96.32%)	320,191,191 (89.30%)	262,166,737 (94.76%)	110,373,205 (93.01%)	113,257,588 (83.73%)	129,887,820 (92.20%)	168,696,853 (86.34%)	253,560,256 (92.50%)
**Hi-C Contacts (% Sequenced Reads; % Unique Reads)**	395,343,162 (63.15% / 79.27%)	194,327,529 (33.50% / 51.91%)	407,127,169 (58.06% / 63.19%)	202,114,957 (77.39% / 89.56%)	240,961,183 (67.20% / 80.68%)	135,149,878 (48.85% / 66.30%)	98,041,793 (82.62% / 90.40%)	74,709,784 (55.23% / 74.71%)	98,990,997 (70.27% / 84.18%)	74,918,770 (38.35% / 66.61%)	192,482,126 (70.22%; 86.76%)
**Inter-chromosomal (% Sequenced Reads; % Unique Reads)**	66,570,270 (10.63% / 13.35%)	36,578,807 (6.31% / 9.77%)	86,740,307 (12.37% / 13.46%)	72,419,139 (27.73% / 32.09%)	67,550,997 (18.84% / 22.62%)	12,407,240 (4.48% / 6.09%)	34,532,138 (29.10% / 31.84%)	28,883,745 (21.35% / 28.88%)	32,069,488 (22.77% / 27.27%)	5,809,147 (2.97% / 5.16%)	59,111,119 (21.56%; 26.64%)
**Intra-chromosomal (% Sequenced Reads; % Unique Reads)**	328,772,892 (52.52% / 65.92%)	157,748,722 (27.19% / 42.14%)	320,386,862 (45.69% / 49.73%)	129,695,818 (49.66% / 57.47%)	173,410,186 (48.36% / 58.06%)	122,742,638 (44.36% / 60.21%)	63,509,655 (53.52% / 58.56%)	45,826,039 (33.88% / 45.82%)	66,921,509 (47.51% / 56.91%)	69,109,623 (35.37% / 61.45%)	133,371,007 (48.65%; 60.12%)
**Short Range (<20Kb) (% Sequenced Reads; % Unique Reads)**	113,928,677 (18.20% / 22.84%)	40,521,898 (6.99% / 10.82%)	95,758,183 (13.66% / 14.86%)	29,548,271 (11.31% / 13.09%)	43,758,213 (12.20% / 14.65%)	63,552,780 (22.97% / 31.18%)	20,021,749 (16.87% / 18.46%)	17,276,086 (12.77% / 17.28%)	20,976,086 (14.89% / 17.84%)	54,160,982 (27.72% / 48.15%)	36,099,196 (13.17%; 16.27%)
**Long Range (>20Kb) (% Sequenced Reads; % Unique Reads)**	214,843,996 (34.32% / 43.08%)	117,226,658 (20.21% / 31.31%)	224,628,009 (32.04% / 34.87%)	100,147,540 (38.34% / 44.38%)	129,651,970 (36.16% / 43.41%)	59,189,746 (21.39% / 29.04%)	43,487,829 (36.65% / 40.10%)	28,549,941 (21.11% / 28.55%)	45,945,389 (32.62% / 39.07%)	14,948,351 (7.65% / 13.29%)	97,271,750 (35.48%; 43.84%)

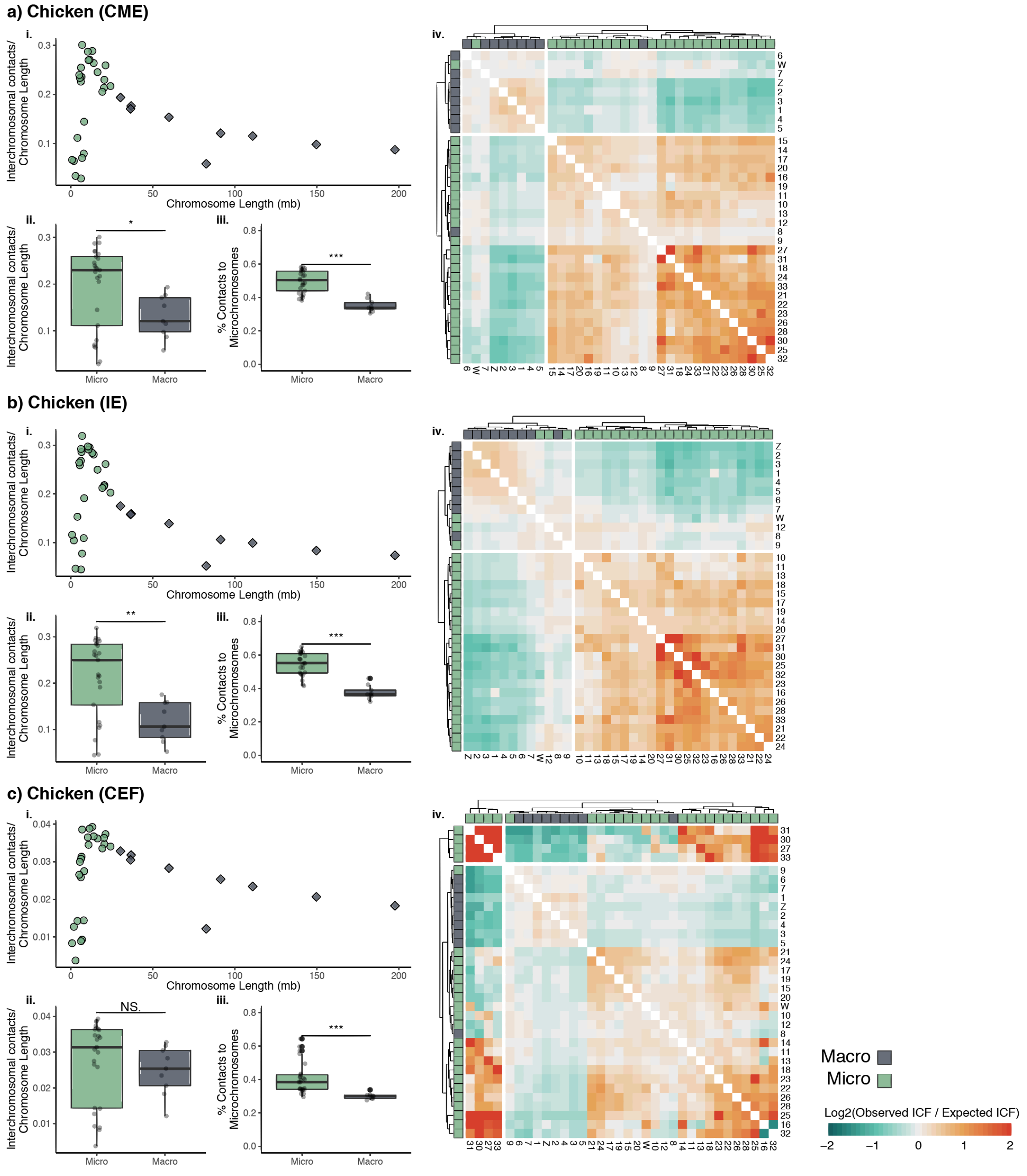

Supplementary Figure 1. **Microchromosomes interactions in three chicken tissues**. Patterns of interchromosomal interaction for chicken mature erythrocyte (CME), immature erythrocyte (IE), and embryonic fibroblast (CEF) cells. a.i-c.i) Sums of interchromosomal contact frequencies per chromosome normalized by chromosome length plotted over chromosome length. a.ii-c.ii) Comparisons of interchromosomal contact frequency normalized by chromosome length for macro and microchromosomes (*: p-value < 0.05, ***: p-value < 0.001, Student's t-test). a.iii-c.iii) Comparison of the proportion of interchromosomal contacts that involve a microchromosome for macrochromosomes and microchromosomes (*** denotes p < 0.001, Student's t-test). a.iv-c.iv) Heatmaps of the ratio of observed to expected interchromosomal contact frequency (ICF) between all chromosome pairs, with hierarchical clustering and chromosome type annotated above and to the left of each heatmap.

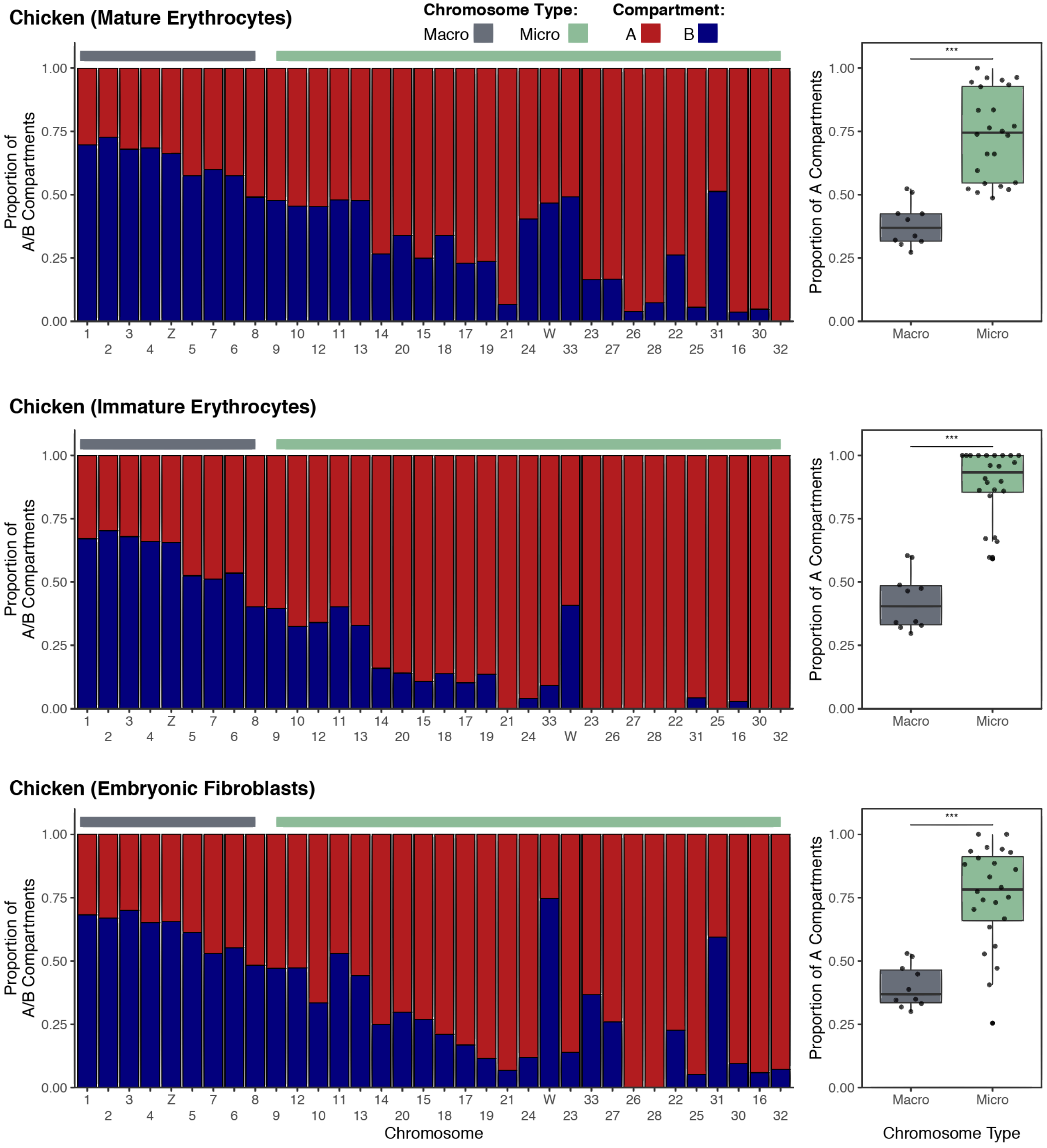

Supplementary Figure 2. Microchromosomes are enriched for the A compartment in all three chicken tissues. Bar plots indicate the proportion of 50 kb bins for each chromosome that were determined to be A (red) and B (blue) compartment. In all tissues, microchromosomes exhibit a higher proportion of A compartment bins than macrochromosomes (boxplots on right; *** denotes $p < 0.001$, Student’s t-test).

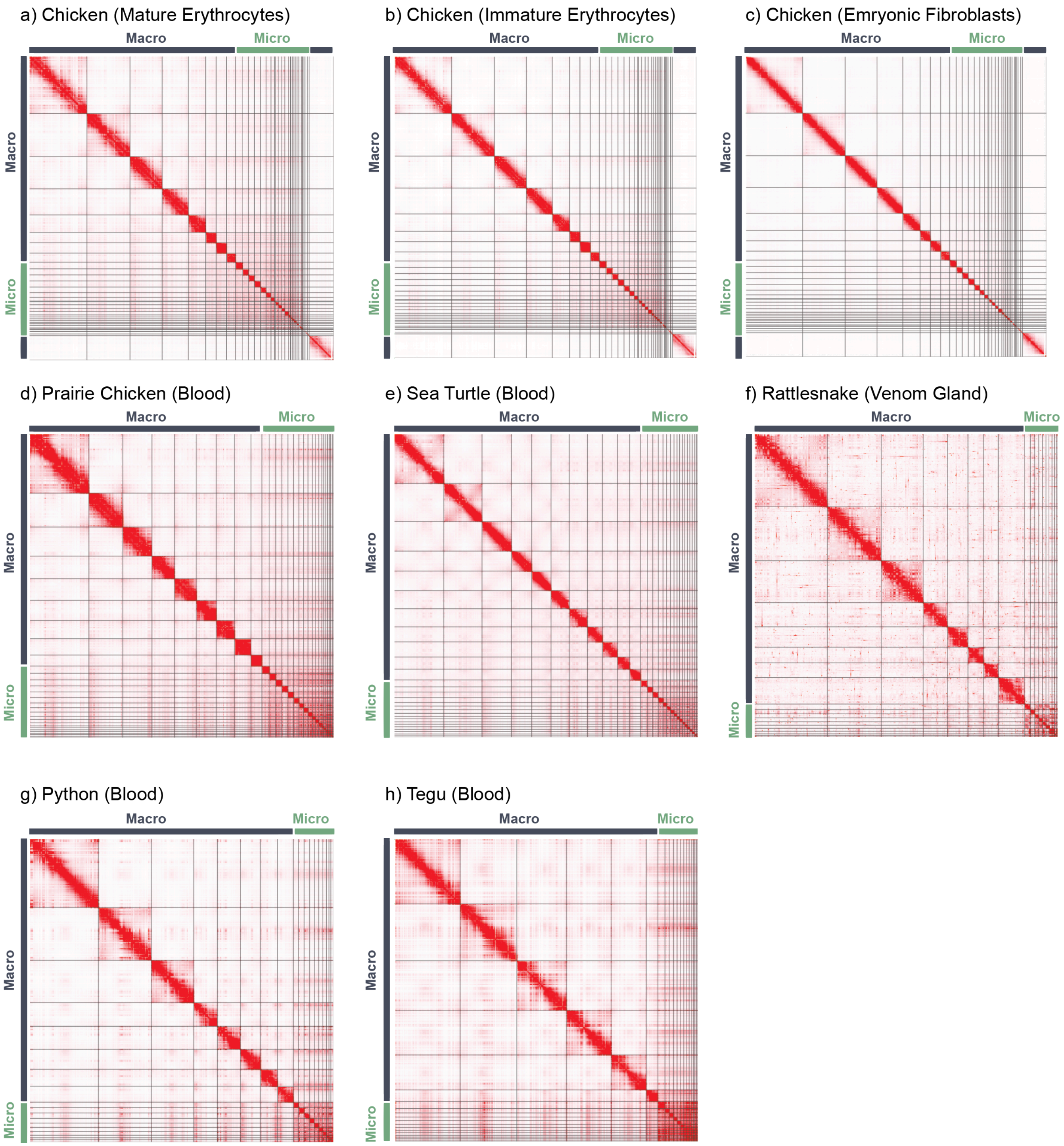

Supplementary Figure 3. Hi-C contact frequency heatmaps at 50kb resolution for all focal species possessing both macrochromosomes and microchromosomes. Darker red indicates higher contact frequency. Chromosome territories are evidenced by defined "blocks" of interaction frequency corresponding to chromosomes that indicate a high degree of self-interaction and lesser degree of interaction with other chromosomes.

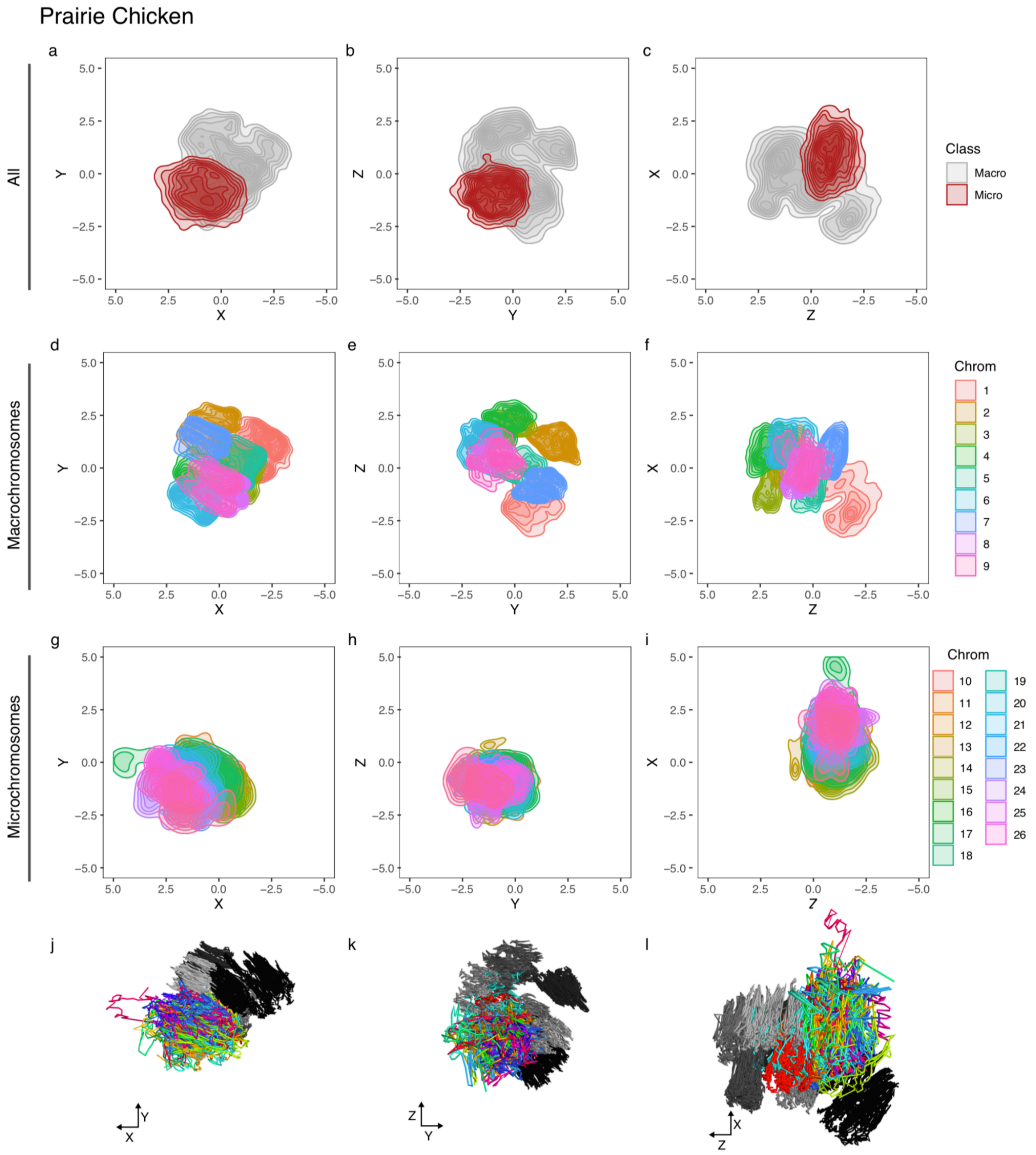

Supplementary Figure 4. 3D interpretation of Prairie Chicken Hi-C interaction data is shown at three distinct orientations (left, center, and right columns), with plots of 2D point density of 3D chromosome models. A-C) 2D point density of all microchromosomes (red) and macrochromosomes (grey). D-F) 2D point density of macrochromosomes only, with each macrochromosome shown as a different color. G-I) 2D point density of microchromosomes only, with each macrochromosome shown as a different color. J-L) 3D models of all chromosomes, with macrochromosomes shown in greyscale and microchromosomes in color.

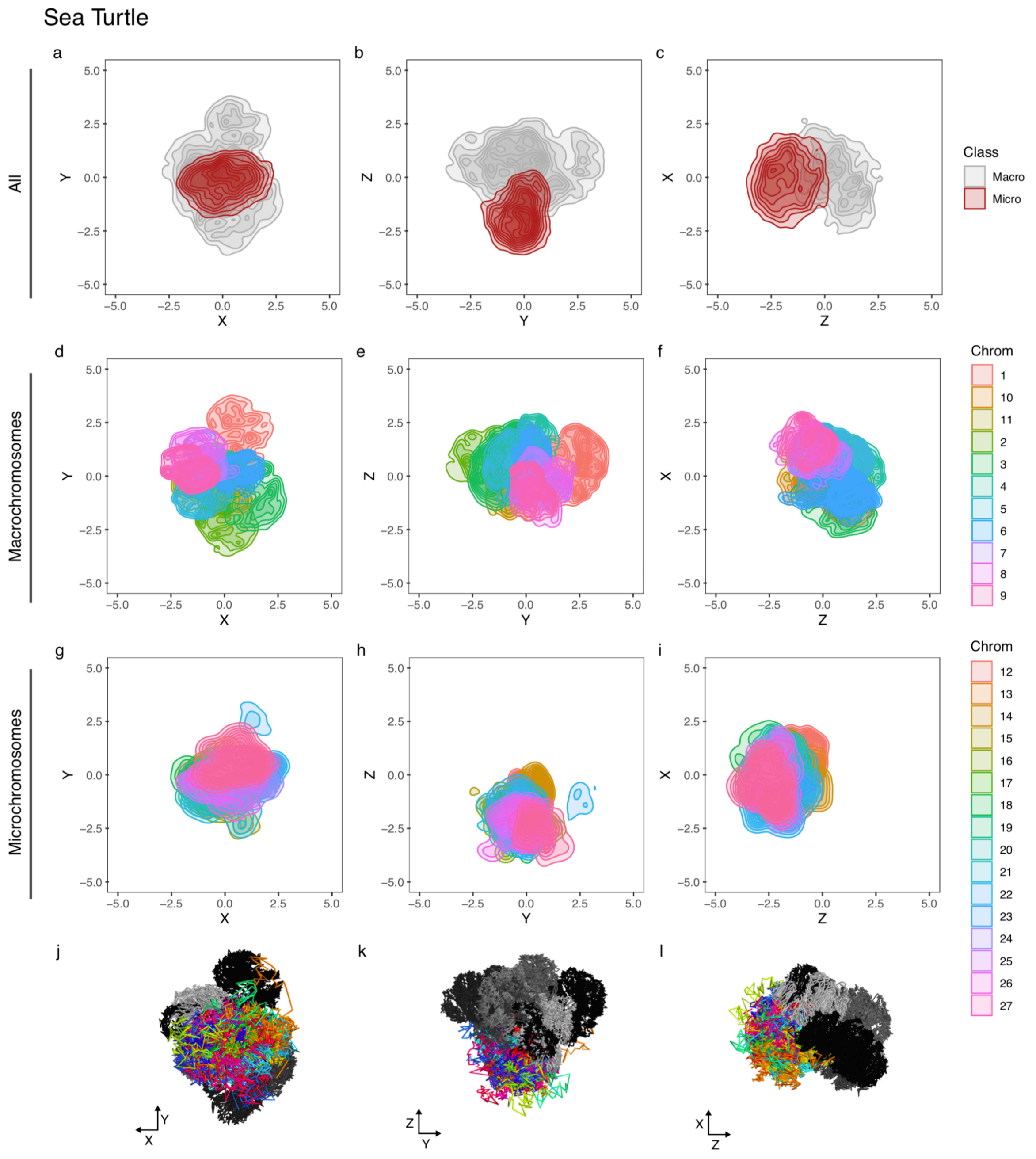

Supplementary Figure 5. 3D interpretation of Sea Turtle Hi-C interaction data is shown at three distinct orientations (left, center, and right columns), with plots of 2D point density of 3D chromosome models. A-C) 2D point density of all microchromosomes (red) and macrochromosomes (grey). D-F) 2D point density of macrochromosomes only, with each macrochromosome shown as a different color. G-I) 2D point density of microchromosomes only, with each macrochromosome shown as a different color. J-L) 3D models of all chromosomes, with macrochromosomes shown in greyscale and microchromosomes in color.

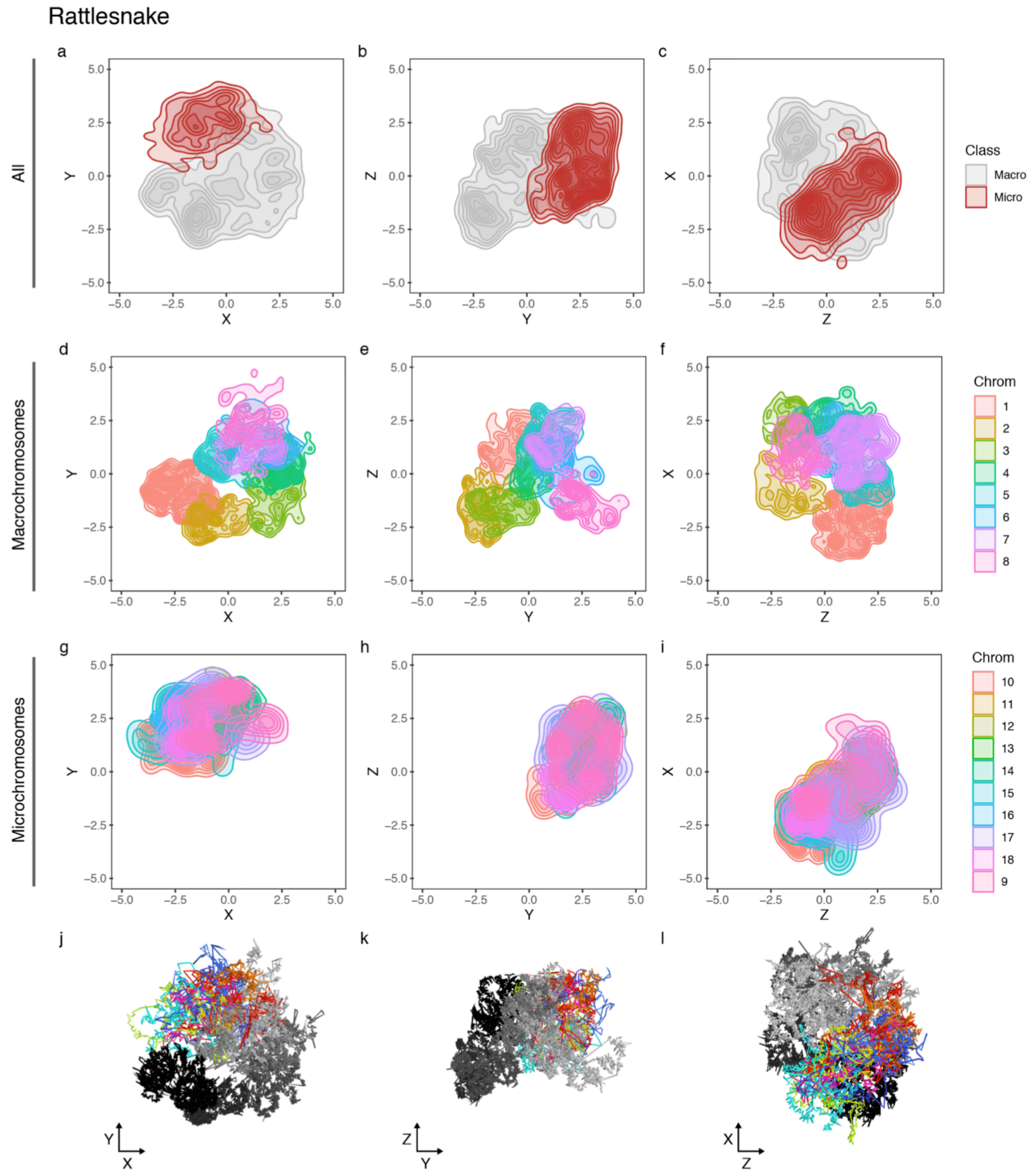

Supplementary Figure 6. 3D interpretation of Rattlesnake Hi-C interaction data is shown at three distinct orientations (left, center, and right columns), with plots of 2D point density of 3D chromosome models. A-C) 2D point density of all microchromosomes (red) and macrochromosomes (grey). D-F) 2D point density of macrochromosomes only, with each macrochromosome shown as a different color. G-I) 2D point density of microchromosomes only, with each macrochromosome shown as a different color. J-L) 3D models of all chromosomes, with macrochromosomes shown in greyscale and microchromosomes in color.

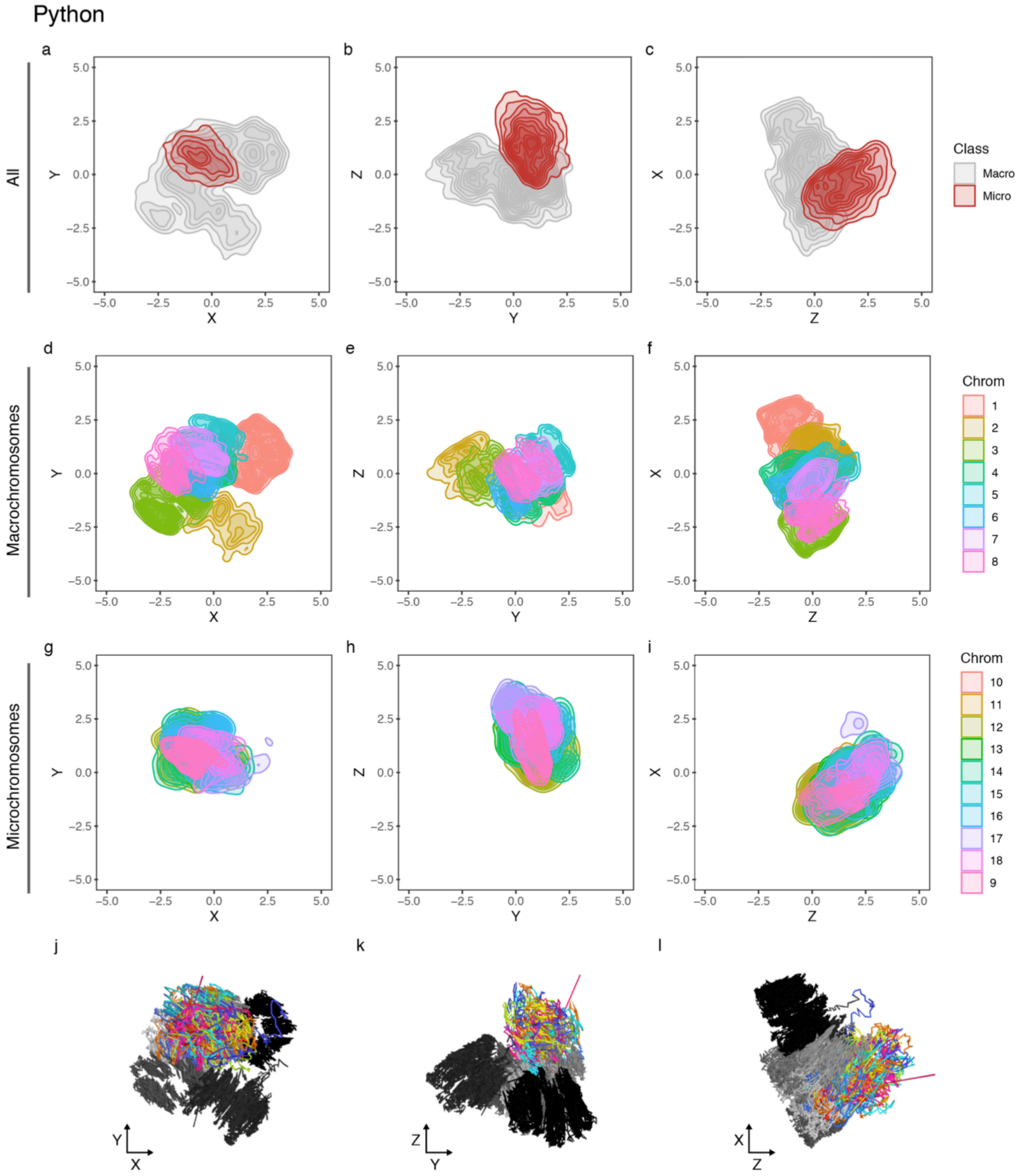

Supplementary Figure 7. 3D interpretation of Python Hi-C interaction data is shown at three distinct orientations (left, center, and right columns), with plots of 2D point density of 3D chromosome models. A-C) 2D point density of all microchromosomes (red) and macrochromosomes (grey). D-F) 2D point density of macrochromosomes only, with each macrochromosome shown as a different color. G-I) 2D point density of microchromosomes only, with each macrochromosome shown as a different color. J-L) 3D models of all chromosomes, with macrochromosomes shown in greyscale and microchromosomes in color.

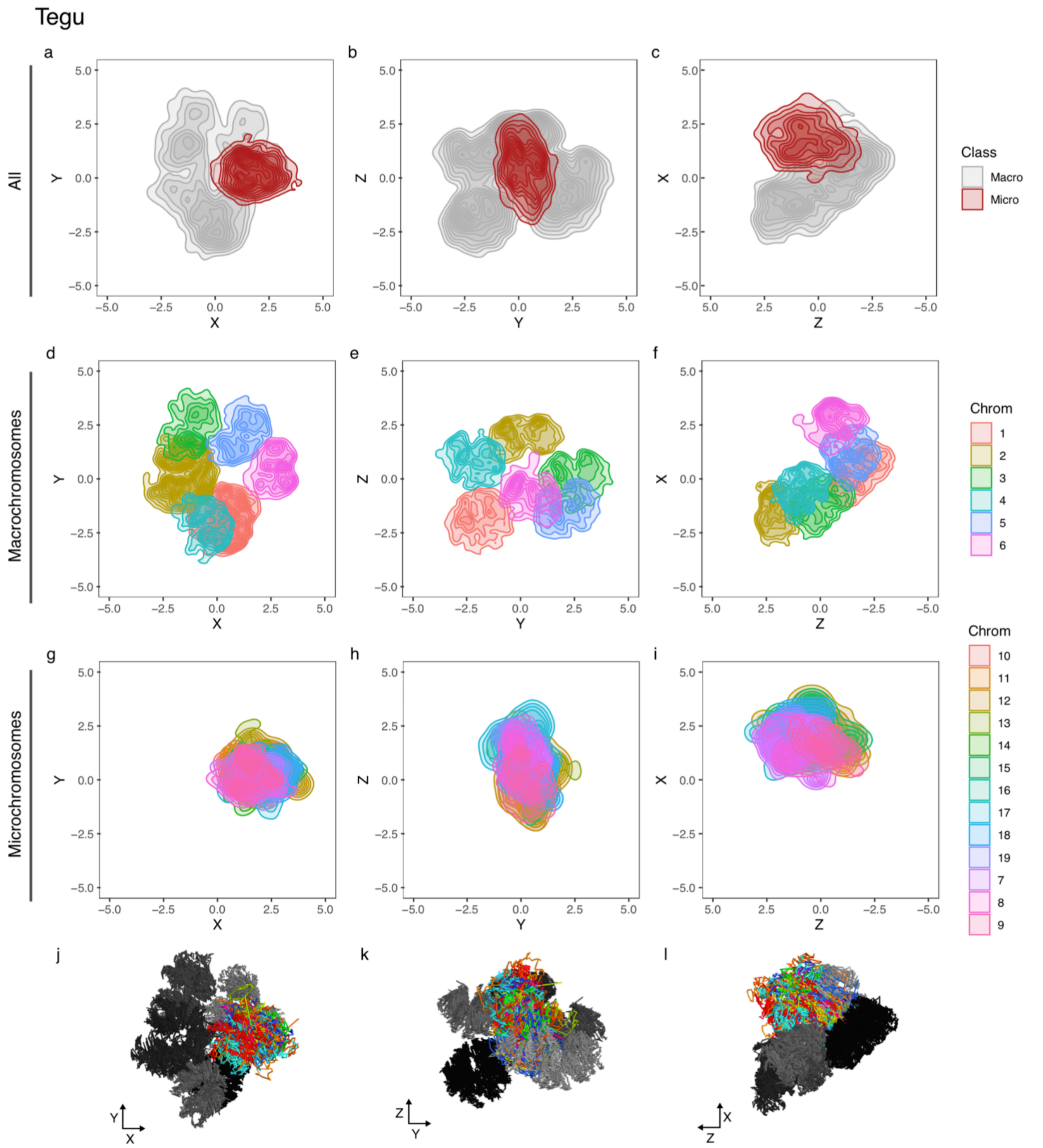

Supplementary Figure 8. 3D interpretation of Tegu Hi-C interaction data is shown at three distinct orientations (left, center, and right columns), with plots of 2D point density of 3D chromosome models. A-C) 2D point density of all microchromosomes (red) and macrochromosomes (grey). D-F) 2D point density of macrochromosomes only, with each macrochromosome shown as a different color. G-I) 2D point density of microchromosomes only, with each macrochromosome shown as a different color. J-L) 3D models of all chromosomes, with macrochromosomes shown in greyscale and microchromosomes in color.

Works Cited:

Castoe TA, de Koning APJ, Hall KT, Card DC, Schield DR, Fujita MK, Ruggiero RP, Degner JF, Daza JM, Gu W, et al. 2013. The Burmese python genome reveals the molecular basis for extreme adaptation in snakes. Proc. Natl. Acad. Sci. [Internet] 110:20645–20650. Available from: http://www.ncbi.nlm.nih.gov/pubmed/24297902

Darrow EM, Huntley MH, Dudchenko O, Stamenova EK, Durand NC, Sun Z, Huang S-C, Sanborn AL, Machol I, Shamim M. 2016. Deletion of DXZ4 on the human inactive X chromosome alters higher-order genome architecture. Proc. Natl. Acad. Sci. 113:E4504–E4512.

Dudchenko O, Batra SS, Omer AD, Nyquist SK, Hoeger M, Durand NC, Shamim MS, Machol I, Lander ES, Aiden AP. 2017. De novo assembly of the Aedes aegypti genome using Hi-C yields chromosome-length scaffolds. Science (80-.). 356:92–95.

Dudchenko O, Shamim MS, Batra S, Durand NC, Musial NT, Mostofa R, Pham M, St Hilaire BG, Yao W, Stamenova E. 2018. The Juicebox Assembly Tools module facilitates de novo assembly of mammalian genomes with chromosome-length scaffolds for under $1000. bioRxiv:254797.

Fishman V, Battulin N, Nuriddinov M, Maslova A, Zlotina A, Strunov A, Chervyakova D, Korablev A, Serov O, Krasikova A. 2018. 3D organization of chicken genome demonstrates evolutionary conservation of topologically associated domains and highlights unique architecture of erythrocytes' chromatin. Nucleic Acids Res. 47:648–665.

Johnson J, Novak BJ, Athrey G, Shapiro B, Phelan R. Whole genome sequence analysis reveals evolutionary history of extinct Heath Hen. Unpublished.

Rao SSP, Huntley MH, Durand NC, Stamenova EK, Bochkov ID, Robinson JT, Sanborn AL, Machol I, Omer AD, Lander ES. 2014. A 3D map of the human genome at kilobase resolution reveals principles of chromatin looping. Cell 159:1665–1680.

Roscito JG, Sameith K, Pippel M, Francoijs K-J, Winkler S, Dahl A, Papoutsoglou G, Myers G, Hiller M. 2018. The genome of the tegu lizard Salvator merianae: combining Illumina, PacBio, and optical mapping data to generate a highly contiguous assembly. Gigascience 7:giy141.

Schield DR, Card DC, Hales NR, Perry BW, Pasquesi GM, Blackmon H, Adams RH, Corbin AB, Smith CF, Ramesh B. 2019. The origins and evolution of chromosomes, dosage compensation, and mechanisms underlying venom regulation in snakes. Genome Res. 29:590–601.

Wang Z, Pascual-Anaya J, Zadissa A, Li W, Niimura Y, Huang Z, Li C, White S, Xiong Z, Fang D. 2013. The draft genomes of soft-shell turtle and green sea turtle yield insights into the development and evolution of the turtle-specific body plan. Nat. Genet. 45:701–706.
